# Endothelium Dependent Vasomotion and *In Vitro* Markers of Endothelial Repair in Patients with Severe Sepsis: An Observational Study

**DOI:** 10.1371/journal.pone.0069499

**Published:** 2013-08-06

**Authors:** Sabrina H. van Ierssel, Emeline M. Van Craenenbroeck, Vicky Y. Hoymans, Christiaan J. Vrints, Viviane M. Conraads, Philippe G. Jorens

**Affiliations:** 1 Department of Critical Care Medicine, Antwerp University Hospital (UZA), University of Antwerp (UA), Edegem, Belgium; 2 Laboratory of Cellular and Molecular Cardiology, Antwerp University Hospital (UZA), University of Antwerp (UA), Edegem, Belgium; 3 Department of Cardiology, Antwerp University Hospital (UZA), University of Antwerp (UA), Edegem, Belgium; Centre Hospitalier Universitaire Vaudois, Switzerland

## Abstract

**Background:**

Outcome in sepsis is mainly defined by the degree of organ failure, for which endothelial dysfunction at the macro- and microvascular level is an important determinant. In this study we evaluated endothelial function in patients with severe sepsis using cellular endothelial markers and *in vivo* assessment of reactive hyperaemia.

**Materials and Methods:**

Patients with severe sepsis (n = 30) and 15 age- and gender- matched healthy volunteers were included in this study. Using flow cytometry, CD34+/KDR+ endothelial progenitor cells (EPC), CD31+ T-cells, and CD31+/CD42b- endothelial microparticles (EMP) were enumerated. Migratory capacity of cultured circulating angiogenic cells (CAC) was assessed *in vitro*. Endothelial function was determined using peripheral arterial tonometry at the fingertip.

**Results:**

In patients with severe sepsis, a lower number of EPC, CD31+ T-cells and a decreased migratory capacity of CAC coincided with a blunted reactive hyperaemia response compared to healthy subjects. The number of EMP, on the other hand, did not differ. The presence of organ failure at admission (SOFA score) was inversely related with the number of CD31+ T-cells. Furthermore, the number of EPC at admission was decreased in patients with progressive organ failure within the first week.

**Conclusion:**

In patients with severe sepsis, *in vivo* measured endothelial dysfunction coincides with lower numbers and reduced function of circulating cells implicated in endothelial repair. Our results suggest that cellular markers of endothelial repair might be valuable in the assessment and evolution of organ dysfunction.

## Background

Sepsis is one of the major indications for admission to the Intensive Care Unit (ICU) [1]. Mortality and morbidity associated with sepsis are mainly determined by the development of organ dysfunction and degree of shock, in which macro- and microvascular dysfunction are key players [2], [3]. Because the endothelium plays a central coordinating role in the regulation of vasomotion, interest in endothelial dysfunction, as a pathophysiological mechanism and possible therapeutic target, is growing [4]. Numerous *in vivo* and *in vitro* techniques to evaluate functional integrity of the endothelium are available.

Cellular mechanisms for endothelial repair, such as endothelial progenitor cells (EPC) and circulating angiogenic cells (CAC), are important for re-endothelialisation and neovascularisation [5]–[7]. Whereas true EPC are considered to differentiate into endothelial cells, CAC support the process of vascular repair through paracrine actions. Observational and experimental studies involving EPC and CAC in sepsis have provided conflicting results [8]–[13]. In selected populations of patients with sepsis, the number of EPC is increased [8]–[10]. On the other hand, administration of lipopolysaccharide in healthy subjects led to a reduction of circulating EPC, which was also seen in an animal model of multi-organ failure [12], [13]. As far as we know, data on the functional capacity of CAC in sepsis are scarce and confined to their proliferative capacity *in vitro* [9], [11].

Endothelial microparticles are small cell membrane particles released from endothelial cells into the circulation upon damage, activation or apoptosis and were, until recently, considered as indicators of these processes [14]. Now their role has been redefined; instead of being mere markers, data suggest they also have various physiological effects [14]. In the context of sepsis, increased as well as unchanged numbers of endothelial microparticles (EMP) have been described [15]–[17]. Moreover in sepsis, circulating microparticles have been linked to the development of shock, but also to an increased sensitivity to serotonin mediated vasoconstriction [15], [17].

Recently, *in vivo*, non-invasive techniques for the evaluation of endothelial function have been developed [18]. Peripheral arterial tonometry (PAT), measured at the fingertips, is increasingly used for the evaluation of endothelium dependent vasodilation in response to reactive hyperaemia. Compared to flow-mediated dilation measured with ultrasound at the level of the brachial artery, PAT is less operator-dependent, reflects local microcirculatory changes and corrects for systemic effects using the contralateral arm as control [18].

In the present study, multi-parametric biomarker evaluation of endothelial function was determined in patients with severe sepsis admitted at the ICU, and compared with a group of healthy subjects. Cellular markers for endothelial damage and repair were quantified and the reactive hyperaemia index (RHI), measured with PAT, was used as an in vivo correlate of microvascular endothelial function. Our aim was to evaluate changes of these parameters in patients with severe sepsis and their possible interrelation, as well as their relation with sepsis severity and the presence or progression of organ failure.

## Materials and Methods

### Patients and Blood sampling

**Figure 1:**
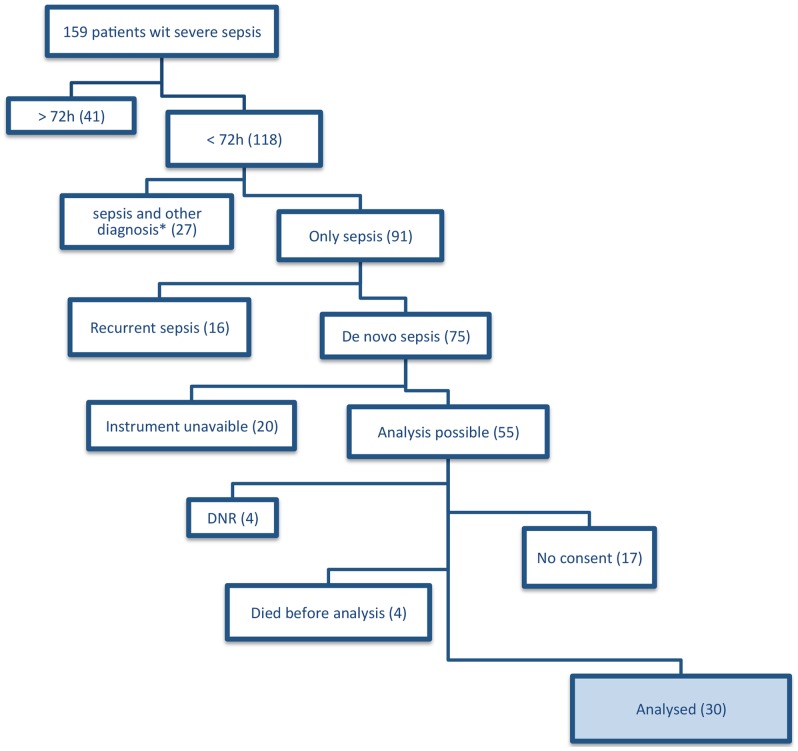
Patient screening. From May 2010 until January 2012, we screened 159 patients with severe sepsis for this study. The major exclusion reason (n = 41) was a diagnosis longer than 72 h before inclusion. This is due to the fact that our center is a tertiary care hospital accepting referrals from ICU's from surrounding centers. In 27 (*) patients there were arguments for cardiogenic or hypovolemic shock or an ischemic event at the time of diagnosis. Four patients had a DNR code at screening, in another 17 consent was refused, 4 died before analysis could be performed, 16 had a recurrent sepsis or a longer standing infection with antibiotic use before diagnosis. Twenty patients could not be analyzed due to technical problems (instrument malfunctioning, unavailability of the instruments).

**Figure 2:**
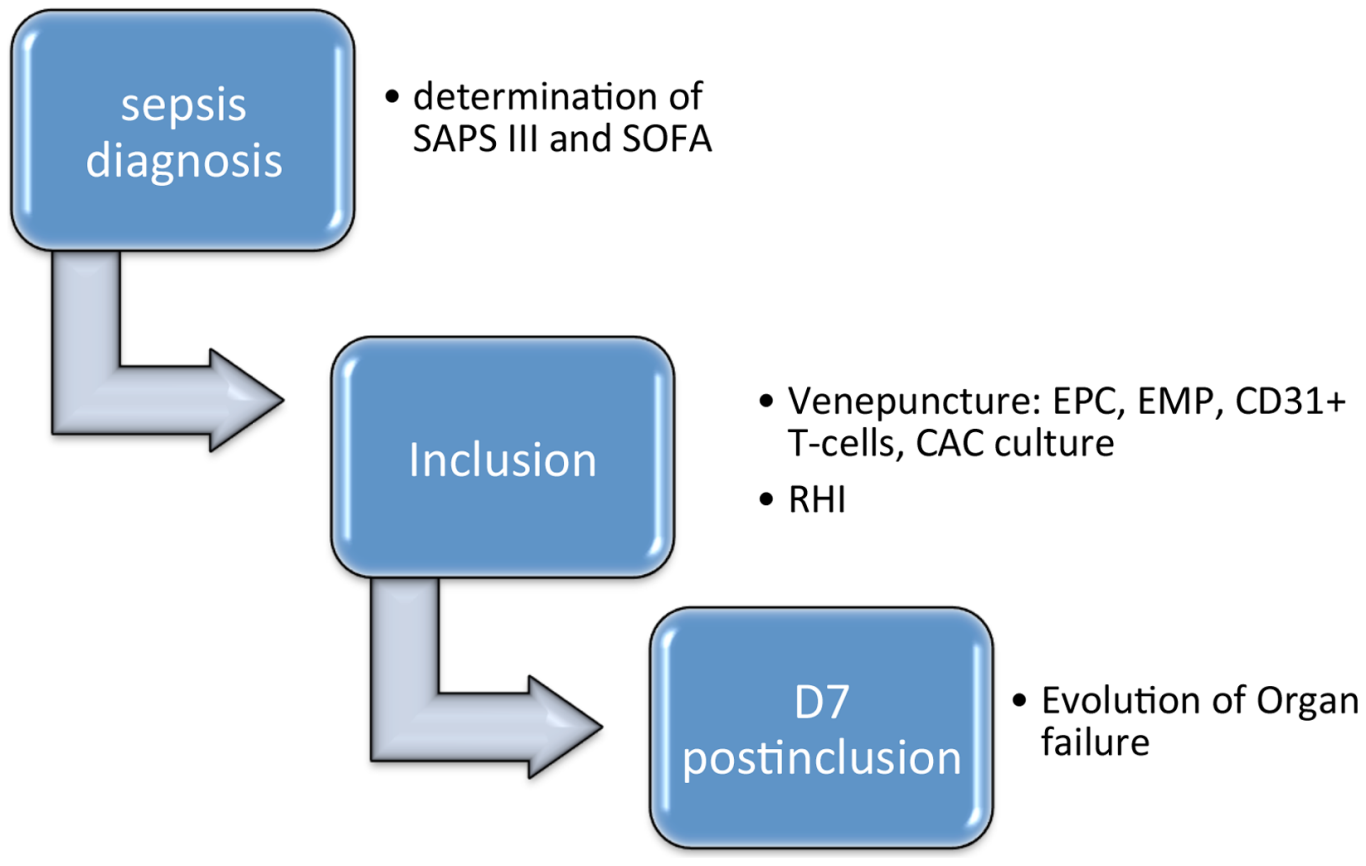
Outline of the study. Flow chart representing the timeline used for measurements and assessment of disease severity.CAC =  circulating angiogenic cell, EMP =  endothelial microparticle, EPC =  endothelial progenitor cell, RHI =  reactive hyperaemia, SAPS =  Simplified Acute Physiology; SOFA =  Sequential of Organ Failure Assessment.

Thirty patients with severe sepsis admitted to the ICU at the Antwerp University Hospital were included between May 2010 and January 2012 (screening outlined in figure 1). We defined severe sepsis according to the criteria of the American College of Chest Physians and Society of Critical Care Medicine developed during the consensus conference on sepsis and organ failure in 1992 [19]. Sepsis, a systemic inflammatory response to infection, with associated organ failure was defined as severe sepsis. This included patients with hypotension despite adequate fluid resuscitation and thus need for vasopressors, i.e. septic shock. Patients had to be older than 18 years, and were only included if they did not have a “do not resuscitate” (DNR) code (see Figure 1). We also excluded patients with a concurrent cardiogenic or hemorrhagic shock and an acute ischemic event present at the moment of severe sepsis diagnosis. Patients who had a longstanding infection with antibiotic use at the diagnosis of sepsis or had a recurrent sepsis were also excluded. For clinical characterisation the Simplified Acute Physiology (SAPS) III scores was calculated on admission. The degree of organ failure was assessed daily at the ICU with the Sequential Organ Failure Assessment (SOFA) score (Figure 2) [20].

Age- and gender-matched healthy subjects (n = 15) were included as a control group. They were free from medication and did not have a history of cardiovascular disease or other chronic conditions. They were evaluated after an overnight fast and having refrained from exercise for at least 24 h.

The Research and Ethics committee of the Antwerp University Hospital approved the study protocol (Belgian number: B30020095394), and written informed consent was obtained from all subjects or their close relatives, corresponding to the principles outlined in the Declaration of Helsinki.

### Flow cytometry

In patients, blood samples were taken from the radial arterial line if available or by peripheral venepuncture. In healthy controls, blood was collected through venepuncture. Samples were collected in acid citrate dextrose vacutainer tubes (BD Medical). The first 3 ml of blood were discarded.

Flow cytometric determination of EPC was executed as previously described with small modifications [21]. Briefly, after FcR-blocking (Miltenyi Biotec), aliquots of 200 μl whole blood were incubated with appropriate concentrations of the following antibodies: PE conjugated KDR (R&Dsystems), PEcy7 conjugated CD34 (BD Biosciences), APC-H7 conjugated CD45 (BD Biosciences). Dead cells and non-nucleated debris were excluded using 7AAD (BD Biosciences) and Syto16 (Molecular Probes, Invitrogen), respectively. A lyse no wash procedure was applied, using ammonium chloride solution (Stemcell Technologies). Samples were assessed immediately on a FACSCantoII (BD Biosciences), and analysis was done using FACSDiva software version 6.1.2 (BD Biosciences). Non-stained and fluorescence-minus-one samples for KDR were used as controls. Gating strategy was based on the ISHAGE protocol, defining EPC as CD34+KDR+CD45dim with low SSC [7]. Results were expressed as the EPC number per 10^5^ CD45 positive mononuclear cells. The coefficient of variation was 18.8±1.2% (n = 7 measurements). In addition, the absolute number (EPC/ml) was determined using the whole blood cell count obtained with the ABX Micros 60 cell counter (Horiba Medicals) within 6 hours of sampling.

Angiogenic T-cells were defined as CD31+ T-cells. [6]. For their detection, 200 μl aliquots were incubated with: FITC conjugated CD31, PerCP conjugated CD3 and APC conjugated CD184 (CXCR4) (all from BD Biosciences). Non-stained samples and fluorescence-minus-one samples for CD31 and CD184 were used as controls. A lyse no wash procedure was used, and results were expressed as a percentage of T-cells. The coefficient of variation is 0.5±0.2%(n = 4 measurements). Furthermore the percentage of CD31+ T-cells expressing CXCR4 (CD184) was calculated.

Determination of the EMP number by flow cytometry on platelet poor plasma (PPP) was performed as previously described [22], [23]. In short, PPP was prepared immediately after blood sampling by centrifugation during 20 minutes at 1550 g twice without acceleration or break. For the detection of EMP, PPP was incubated with CD31-PE and CD42b-FITC (BD Biosciences) and immediately analysed. The upper detection limit of the microparticle gate on FSC and SSC was established using Fluoresbrite YG 1 µm calibration size beads (Polysciences). EMP were defined as particles smaller than 1 µm that were CD31-positive and CD42b-negative. All samples were measured in duplicate (coefficient of variation 4.7±0.9%). Due to technical problems the number of EMP could not be determined in 4 patients.

### Functional capacity of circulating angiogenic cells

CAC were obtained by isolating peripheral blood mononuclear cells (PBMC) using lymphocyte separation medium (MP biomedicals) density centrifugation. It is known that these isolates contain a high percentage of contaminating granulocytes, up to 50%, in sepsis patients [24]. We were able to eliminate these cells successfully by using Rosettesep human granulocyte depletion cocktail (Stem Cell technologies, own data not shown). PBMC were cultured in endothelial growth medium (EGM2-MV, Lonza) supplemented with 20% foetal bovine serum (Sigma Aldrich) on fibronectin (Roche) coated 24 well plates during 1 week as previously described [21]. We did obtain an adequate CAC culture in 22 severe sepsis patients and 14 controls.

The migration capacity of these cells to stromal cell derived factor 1α (SDF-1α, 100 ng/ml, R&D systems) and vascular endothelial growth factor (VEGF, 50 ng/ml, R&D systems) was determined in 5 μm transwells (Corning Costar). All measures were performed in duplicate (coefficient of variation was 4.91±0.75%). We could reliably evaluate migration capacity in 17 patients with severe sepsis and 14 healthy controls.

### Reactive hyperaemia

Endothelial mediated vasomotion was determined using peripheral arterial tonometry (PAT) at the finger after upper-arm occlusion (endoPAT, Itamar). The measurement was performed 30 minutes after blood sampling as previously described [23]. Healthy volunteers were analysed in a quiet room after acclimatization. Patients with sepsis were evaluated bedside, and had to be haemodynamically stable, meaning no fluid bolus or changes in vasopressors or inotropic doses, for at least 30 minutes. The RHI was calculated with the Itamar software version 3.2.4. The coefficient of variation was 9.0±2.4% for measurements on different study days in healthy volunteers (n = 12). We assessed reactive hyperaemia in 20 severe sepsis patients, and all healthy controls. In severe sepsis patients endoPAT measurement was complicated by technical problems (n = 3), involuntary movements of the hands (n = 1), bilateral axillary lymphadenectomy (n = 1), necrotising fasciitis involving the hand (n = 1), important coagulopathy (n = 2) or suboptimal signal (n = 2).

### Statistics

Statistical analysis was performed using SPSS version 20.0.00. Results are expressed as mean ± standard error of the mean (SEM). Normality of continuous variables was evaluated using Kolmogorov-Smirnov test with Lilliefors correction. Groups were compared using independent T-test or Mann-Whitney U-test with or without exact testing, where appropriate. For the comparison of frequencies the X^2^-test was used. Linear relation was examined using Pearson, Spearman or partial correlation correcting for age wherever applicable.

## Results

### Study population

We included 30 patients with sepsis associated organ failure, of which 23 (76%) fulfilled the defining criteria for septic shock. The baseline characteristics of the sepsis group are presented in Table 1. Time between sepsis diagnosis and blood sampling was 31.8±3.3 hours (mean ± SEM). Since two patients were put on a do not resuscitate (DNR) code after inclusion, hospital mortality was only assessed in 28 patients. Four patients died; only one of them died within 28 days after sepsis diagnosis. Healthy controls (n = 15) and severe sepsis patients were age- and gender-matched (Table 2).

**Table 1:** Baseline characteristics of the severe sepsis patients (n = 30).

Age (years)	63.0±2.9	Infection	
BMI	26.2±0.9	- Pneumonia	11 (37%)
Male	20 (67%)	- Intra-abdominal	8 (27%)
Co-morbidity		- Skin and soft tissue	4 (13%)
- Obesity	5 (17%)	- Urosepsis	2 (7%)
- Cardiovascular disease	12 (40%)	- Other	5 (17%)
- Hyperlipidaemia	5 (17%)	Bacteraemia	9 (30%)
- Diabetes	5 (17%)	Microbiological confirmation	25 (83%)
- Malignancy	6 (20%)	Treatment	
- Chronic kidney disease	2 (7%)	Adequate antibiotics (n = 25)	19 (76%)
- Inflammatory disease	2 (7%)	- Surgery	16 (53%)
- Immunosuppressed	6 (20%)	- Mechanical ventilation	17 (57%)
Chronic medication		- Renal replacement therapy	5 (17%)
- ACE-inhibitor or sartan	7 (23%)	- Corticosteroids	8 (27%)
- Statins	5 (17%)	- Immunoglobulins	3 (10%)
- Corticosteroids	4 (13%)	Vasopressors/Inotropics number (mean max dose ± SEM)	
Sepsis Severity		- Norepinephrine (μg/kg/min)	21 (0.35±0.04)
- SAPSIII	61.7±1.9	- Adrenaline (μg/kg/min)	2 (0.16±0.12)
- SOFA D0	7.8±0.6	- Dobutamine (μg/kg/min)	5 (4.5±0.7)
Clinical parameters		- Vasopressin (IE/h)	6 (2.3±0.6)
- MAP (mmHg)	79.2±1.6	- Milrinone (μg/kg/min)	2 (0.21±0.04)
- MII	23.3±8.5	ICU stay (h)	286±69
- VDI	8.1±1.8	Hospital stay (d)	41±8
		Hospital mortality (n = 28)	4 (14%)

BMI = body mass index; ICU =  intensive care unit; MAP =  Mean arterial pressure; MII =  Modified Inotropic Index; POD = persistent organ failure; SAPS =  Simplified Acute Physiology; SOFA =  Sequential of Organ Failure Assessment; VDI =  Vasopressor Dependency Index; h =  hour; d =  day.

Data presented as mean ± SEM or as number (percentage).

**Table 2:** Comparison of markers of endothelial function in severe sepsis with those in healthy volunteers.

Variable (n*)	Severe Sepsis	Healthy volunteers	p-value
Age in years (30,15)	63.0±2.9	56.5±3.5	0.187
BMI (30,15)	26.2±0.9	24.0±0.7	0.105
Male (30,15)	20 (67%)	8 (53%)	0.384
WBC x10^6^/ml (30,15)	14.9±1.4	5.3±0.3	<0.001
PBMC x10^6^/ml (28,15)	1.3±0.2	2.0±0.1	<0.001
CD34+ cells/ml (28,15)	2225±532	2461±435	0.019
CD34/10^5^PBMC (30,15)	190.1±39.5	131.5±26.3	0.942
EPC/ml (28,15)	213.1±45.1	452.0±107.2	0.001
EPC/10^5^ PBMC (30,15)	22.6±3.9	24.1±6.3	0.273
%CD31+ T-cells (30,15)	35.1±2.5	43.1±2.6	0.050
%CD184+CD31+ T-cells (30,15)	91.8±1.1	83.0±1.8	<0.001
EMP/μl (26,15)	203.3±20.3	205.2±23.5	0.739
%CAC migration (17,14)	32.5±3.5	45.3±4.9	0.038
RHI (20,15)	1.88±0.11	2.41±0.14	0.004

BMI =  body mass index; CAC =  circulating angiogenic cell, EPC =  endothelial progenitor cell; EMP =  endothelial microparticle; PBMC =  peripheral blood mononuclear cells; RHI = reactive hyperaemia index, WBC = White blood cell count.

Data presented as mea n± SEM or as number (percentage).

* n =  indicates number of patients with severe sepsis and healthy volunteers analysed.

### *In vivo* endothelial function and cellular markers

Results are summarized in Table 2. Endothelial dependent vasomotion, assessed with RHI, was significantly impaired in sepsis patients. There was no difference in RHI between patients on vasopressors and those without (1.85±0.14 vs. 1.95±0.14 respectively, p = 0.672). The Vasopressor Dependency Index (VDI) and Modified Inotropic Index (MII) were calculated to determine vasopressor need at the time of blood sampling and at the moment RHI was measured [25]. RHI was not related to the need for vasopressors at the time of assessment (p = 0.805, 0.858, 0.274 and r = 0.059, −0.043, −0.317 for RHI and respectively norepinephrine dose, modified inotropic index, vasopressor dependency index).

We found a decreased absolute number of CD34+cells and EPC in patients with sepsis. The percentage of CD31+ T-cells in sepsis patients was significantly lower, but the percentage of CD31+ T-cells expressing CD184 was higher. CAC demonstrated impaired migration capacity to SDF-1α and VEGF. Circulating EMP numbers were not different, comparing both groups. We previously demonstrated that lipid solutions used *in vivo* can interfere with the number of EMP detected by flow cytometry [23]. For this reason we compared patients treated with and without propofol or total parenteral nutrition, both of which are known to cause a lipid overload. We could not find a significant difference in EMP number between these groups (189.5±24.8 and 213.5±30.5 for severe sepsis with and without propofol or TPN respectively, p = 0.569). Taken together, these findings point at an impaired endothelial repair capacity in patients with severe sepsis. On the other hand, there was no relation between any of the cellular markers and RHI (p = 0.834, 0.551, 0.448 and r = 0.052, −0.142, 0.185 for RHI and absolute number of EPC, CD31+ T cells and EMP; n = 20).

In severe sepsis patients we found an inverse relation between de reactive hyperaemia measured and the CRP level detected in plasma at inclusion (r = −0.563, p = 0.010). We did not find a relation between CRP and other measured markers of endothelial dysfunction.

### Relation of endothelial dysfunction and sepsis severity and organ failure

We quantified SAPS III scores at admission to evaluate disease severity. There was a strong inverse correlation between the percentage of CD31+ T-cells and SAPS III score (r = −0.468, p = 0.009), however, after correction for age, this relation was no longer significant (partial correlation r = −0.190, p = 0.334). Indeed, CD31+ T-cells correlated significantly with age in sepsis as well as in healthy volunteers (r = −0.639 and −0.860 respectively, p<0.001 in both). We could not find a relation of SAPS III scores with RHI, EPC or EMP numbers, nor with the migratory capacity of CAC.

SOFA score at admission also correlated inversely with CD31+ T-cells (r = −0.372 and p = 0.047). In contrast to the SAPS III score correction for age strengthened the relation between SOFA score and CD31+ T-cells (r = −0.477, p = 0.010). For other cellular markers and RHI, the relation with the severity of organ failure was absent.

Patients in whom the SOFA-score increased (further progression of organ failure, n = 12) during the first week after study inclusion, demonstrated a lower absolute number of EPC and increased CD184 expression on angiogenic T-cells (101.3±33.7 vs. 285.4±66.0 and 94.6±1.3 vs. 89.9±1.4 in increased vs. stable or decreasing SOFA score, p = 0.021 and p = 0.014 respectively) (Table 3). There was also a trend towards lower migration capacity in this group of patients (24.0±6.0 vs. 37.1±3.8 in increased vs. stable or decreasing SOFA score, p = 0.088; Table 3).

**Table 3:** Comparison of markers of endothelial function between patients with and those without progression of organ failure in the first week.

Variable (n*)	Progression of organ failure	No progression of organ failure	p-value
SAPS III (12, 18)	66.3±3.1	58.1±1.7	*0.018*
SOFA (12, 18)	*8.5*±*0.9*	*7.2*±*0.8*	0.298
PBMC x10^6^/ml (11,17)	0.9±0.2	*1.5*±*0.4*	*0.239*
CD34+ cells/ml (11,17)	*1258*±*590*	*2850*±*765*	*0.109*
CD34/10^5^PBMC (12, 18)	*175.3*±*56.2*	*200.0*±*55.3*	0.691
EPC/ml (11,17)	*101.3*±*33.7*	*285.4*±*66.0*	*0.021*
EPC/10^5^ PBMC (12, 18)	23.2±8.0	*22.4*±*3.9*	*0.551*
%CD31+ T-cells (12, 18)	*30.9*±*3.7*	37.8±3.3	*0.175*
%CD184+CD31+ T-cells (12, 18)	94.6±1.3	*89.9*±*1.4*	*0.014*
EMP/μl (10,16)	*161.0*±*27.2*	*229.8*±*26.7*	*0.099*
%CAC migration (6,11)	*24.0*±*6.0*	*37.1*±*3.8*	*0.088*
RHI (7,13)	*1.78*±*0.23*	*1.93*±*0.10*	*0.275*

CAC =  circulating angiogenic cell, EPC =  endothelial progenitor cell; EMP =  endothelial microparticle; PBMC =  peripheral blood mononuclear cells; RHI = reactive hyperaemia index, SAPS =  Simplified Acute Physiology; SOFA =  Sequential of Organ Failure Assessment.

Data presented as mean ± SEM or as number (percentage).

* n =  number of patients analysed with progression and no progression of organ failure respectively.

## Discussion

Vascular dysfunction plays a key role in the development of organ failure, which strongly determines outcome in sepsis patients. In this study we evaluated cellular markers of endothelial function and *in vivo* reactive hyperaemia in a group of 30 patients with severe sepsis, and compared results to a group of healthy volunteers. We found that severe sepsis leads to *in vivo* endothelial dysfunction, which coincides with reduced cellular endothelial repair capacity. Furthermore, sepsis patients with progressive organ dysfunction had lower absolute EPC and an impaired migratory capacity of CAC, despite a higher percentage of CD184 expressing angiogenic T-cells.

### Impaired endothelial repair and vascular dysfunction in severe sepsis

In patients with cardiovascular disease, numbers of circulating CD34+KDR+ EPC are inversely related to vascular damage [26]. We therefore chose this phenotype for the enumeration of EPC in the present study. Similar to cardiovascular conditions, associated with endothelial dysfunction, our results indicate a lower absolute number of EPC in patients with severe sepsis. This is in accordance with experimental studies of sepsis [12], [13], but on the other hand some previous observational studies have found an increased percentage of circulating EPC [8]–[10]. These opposing results may be explained by several differences between studies, more specifically with regard to the population of interest and the specific EPC-phenotype that was measured. First of all, until now, observational studies performed flow cytometry on preselected PBMC or CD34+ cells, different from direct measurements in whole blood in the present study and the experimental setups. The expression of EPC as a percentage of PBMC, as was done in the former studies, will not adequately reflect absolute EPC numbers since PBMC are decreased in sepsis [24]. Furthermore, these observational studies were performed in a preselected patient population, excluding various co-morbidities, and therefore do not reflect a clinical setting.

Circulating angiogenic cells, including CD31+ T-cells and formerly termed early outgrowth EPC, are increasingly recognized as major players in endothelial repair [6]. Our study is the first observational study in severe sepsis patients taking both the number of CD31+ T-cells and the migratory capacity of CAC into account. We found a decreased percentage of angiogenic T-cells in severe sepsis with an increased expression of CD184, the receptor for SDF-1 α. The latter confirms the previous observation of augmented CD184 expression on lymphocytes in sepsis [27]. However, in that study, increased expression of CD184 coincided with a better migration capacity of these cells to SDF-1α. We, on the other hand, found impaired migration of CAC to SDF-1α and VEGF in sepsis, which was also seen in the experimental animal study of Luo et al [13]. These findings suggests that, besides the occurrence of endothelial damage and endothelial cell apoptosis, which are well known processes in sepsis [4], the concurrent impaired vascular repair capacity may contribute to endothelial dysfunction.

In our study, circulating EMP, defined as CD31+CD42b-, did not differ between severe sepsis patients and healthy volunteers. While EMP were previously seen as markers of endothelial damage and/or activation, a growing number of investigators has shown that they may also have pathophysiological effects [14]. In the present study, no relations between EMP numbers at admission and sepsis severity, nor the presence of organ failure, were found, thereby confirming the results of Soriano et al [28].

Endothelial dysfunction in patients with hemodynamic instability in general, and sepsis in particular, is currently accepted as a major contributor to disease severity and progression to organ failure [4]. We confirmed the findings of a blunted endothelial dependent vasomotion, as was found by others [29], [30]. Our severe sepsis population had RHI values slightly higher than those obtained in the study of Davis et al (1.88 vs. 1.57, respectively). This could be due to differences in the technique used, e.g. upper versus lower arm occlusion.

### Endothelial markers as a measure and predictor of organ dysfunction in severe sepsis

A novel finding of our study is that the degree of organ failure, expressed by a higher SOFA score, is inversely related to circulating numbers of CD31+ T-cells. There was also a trend towards impaired migratory capacity of CAC in those patients who had a further progression of organ failure during the first week after inclusion. These new findings indicate that CAC dysfunction might play a role in progression of organ dysfunction in severe sepsis.

Current treatment options are insufficient to specifically tackle the underlying processes that lead to organ failure in sepsis, e.g. endothelial dysfunction. One of the main difficulties is that the clinical evolution of sepsis towards the state of organ dysfunction and shock is difficult to predict. Due to the central role of endothelial dysfunction in the development of organ failure, endothelial markers are of particular interest.

The present findings suggest that lower numbers of circulating EPC may indicate impending clinical deterioration. Although our study was underpowered to firmly establish the predictive value of cellular markers of endothelial repair and reactive hyperaemia regarding organ failure progression we do believe that the current novel results warrant further investigation.

Despite the novel findings, our study has limitations. First, we included a rather heterogeneous group of sepsis patients that however, were felt to reflect clinical reality. Furthermore, the choice of including a gender and age-matched healthy control group as a comparator is debatable. For this study population several different control groups would have been possible, all of them having their own benefits and limitations. We chose gender and age-matched healthy subjects without co morbidities as a control group, since controlling for all possible interfering factors would imply very large study and control populations. For the same reason regression analysis, correcting for factors that may act as confounders was not possible. We therefore consider the present study as an important first exploratory investigation, comparing various cellular markers of endothelial function and relating them to disease severity. Second we only provide a one-time measurement instead of a time-course study due to practical issues, since multiparameter analysis using fresh blood samples limits the frequency of analysis. In particular for EMP, which are very short living circulating particles, timing could be of great importance. At this moment there is no study available on the evolution of EMP early, within 48 h of diagnosis, in sepsis. We also had a low mortality rate in our study that can be due to selection bias, we did include patients somewhat later in their disease course, and patients on a DNR code at that time were excluded. Furthermore the need for vasopressors is left to the judgement of the treating physician. Last, we did not obtain RHI and biomarker analysis in 100% of cases due to multiple reasons inherent to our severely ill patient population (see method section). However these subpopulations were representative of the overall severe sepsis population we analysed as regards to their age, gender, disease severity (SAPSIII and SOFA scores) and co-morbidities.

## Conclusion

In this observational study of severe sepsis patients we found significantly lower numbers of cellular markers of vascular repair, together with a blunted reactive hyperaemia. Furthermore our results indicate that cellular markers of endothelial repair and RHI could be valuable for the assessment and prognosis of organ dysfunction in patients with severe sepsis. Further confirmation of our findings in more sizable, multi-centre studies, is warranted.
